# Studying dietary intake in daily life through multilevel two-part modelling: a novel analytical approach and its practical application

**DOI:** 10.1186/s12966-021-01187-8

**Published:** 2021-09-27

**Authors:** Alea Ruf, Andreas B. Neubauer, Ulrich Ebner-Priemer, Andreas Reif, Silke Matura

**Affiliations:** 1 Department of Psychiatry, Psychosomatic Medicine and Psychotherapy, University Hospital, Goethe University, Heinrich-Hoffmann-Straße 10, 60528 Frankfurt am Main, Germany; 2 DIPF | Leibniz Institute for Research and Information in Education, Frankfurt am Main, Germany; 3 Center for Research on Individual Development and Adaptive Education of Children at Risk (IDeA), Frankfurt am Main, Germany; 4 Mental mHealth Lab, Institute of Sports and Sports Science, Karlsruhe Institute of Technology (KIT), Karlsruhe, Germany; 5 Department of Psychiatry and Psychotherapy, Central Institute of Mental Health, Medical Faculty Mannheim, Heidelberg University, Mannheim, Germany

**Keywords:** Multilevel two-part modelling, Semicontinuous, Longitudinal, Dietary intake, Ecological momentary assessment, R, Brms

## Abstract

**Background:**

Understanding which factors influence dietary intake, particularly in daily life, is crucial given the impact diet has on physical as well as mental health. However, a factor might influence whether but not how much an individual eats and vice versa or a factor’s importance may differ across these two facets. Distinguishing between these two facets, hence, studying dietary intake as a dual process is conceptually promising and not only allows further insights, but also solves a statistical issue. When assessing the association between a predictor (e.g. momentary affect) and subsequent dietary intake in daily life through ecological momentary assessment (EMA), the outcome variable (e.g. energy intake within a predefined time-interval) is semicontinuous. That is, one part is equal to zero (i.e. no dietary intake occurred) and the other contains right-skewed positive values (i.e. dietary intake occurred, but often only small amounts are consumed). However, linear multilevel modelling which is commonly used for EMA data to account for repeated measures within individuals cannot be applied to semicontinuous outcomes. A highly informative statistical approach for semicontinuous outcomes is multilevel two-part modelling which treats the outcome as generated by a dual process, combining a multilevel logistic/probit regression for zeros and a multilevel (generalized) linear regression for nonzero values.

**Methods:**

A multilevel two-part model combining a multilevel logistic regression to predict whether an individual eats and a multilevel gamma regression to predict how much is eaten, if an individual eats, is proposed. Its general implementation in R, a widely used and freely available statistical software, using the R-package brms is described. To illustrate its practical application, the analytical approach is applied exemplary to data from the Eat2beNICE-APPetite-study.

**Results:**

Results highlight that the proposed multilevel two-part model reveals process-specific associations which cannot be detected through traditional multilevel modelling.

**Conclusions:**

This paper is the first to introduce multilevel two-part modelling as a novel analytical approach to study dietary intake in daily life. Studying dietary intake through multilevel two-part modelling is conceptually as well as methodologically promising. Findings can be translated to tailored nutritional interventions targeting either the occurrence or the amount of dietary intake.

**Supplementary Information:**

The online version contains supplementary material available at 10.1186/s12966-021-01187-8.

## Background

Which factors influence whether an individual eats? Which factors influence how much an individual eats? These two questions might be answered differently. For instance, a study found that inhibitory control predicted how much individuals snacked, whereas it did not predict whether individuals snacked [1]. These findings emphasize the dual character of dietary intake. Understanding which factors drive an individual to eat as well as which factors influence how much an individual eats, particularly in daily life, is crucial given the impact diet has on physical as well as mental health.

Diet is a repeated-occurrence health behaviour which is performed multiple times per day [2]. Studying eating behaviour through ecological momentary assessment (EMA) several times a day in natural environments when and “where the action takes place” [3] is a promising and increasingly popular approach [4, 5]. Dietary intake is influenced by a variety of dynamic factors and their interactions [6] which cannot be replicated reliably in a laboratory setting, highlighting the need for EMA.

Studying dietary intake as a dual process in daily life is conceptually promising and not only allows novel insights, but also solves a statistical issue.

### Distributional characteristic of dietary data in EMA studies

**Fig. 1:**
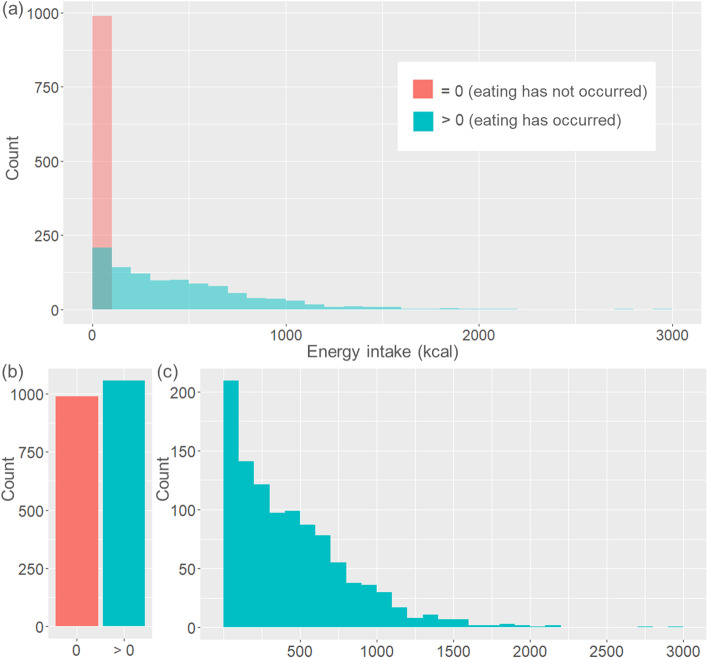
Illustration of distributional characteristics of a semicontinuous dietary outcome (exemplary: Energy intake in kilocalories [kcal] of the data used in this paper), **a** overall distribution containing a large number of zeros as well as right-skewed positive values, **b** distribution of zeros vs. nonzero values, **c** distribution of the right-skewed positive values

EMA studies allow investigating whether individual and/or situational factors (e.g. momentary affect) assessed multiple times per day predict dietary intake (e.g. energy/sugar/fat intake) within a subsequent predefined time-interval (e.g. within the next 2 h). However, dietary intake typically does not occur within each predefined time-interval (e.g. no intake in 46% of 2-h-time-intervals [7]) or only a small amount is consumed (e.g. a snack). This results in an outcome that is zero-inflated (i.e. contains a large proportion of zeros) and right-skewed (i.e. contains a large proportion of small positive values concentrated on the left of the distribution) (see Fig. 1). This type of data is often referred to as semicontinuous.

### Traditional statistical approach for EMA data

A common statistical approach to analyse EMA data is linear multilevel modelling (also known as linear mixed or linear hierarchical modelling). It accounts for dependency among longitudinal data due to repeated measures within the same participant and allows studying effects on the level of moments (within-person fluctuations) and individuals (between-person differences). However, traditional linear multilevel modelling cannot be applied to semicontinuous outcomes, as the assumption of normally distributed residuals is likely violated.^1^

Using traditional linear multilevel models without accounting for the large proportion of zeros can lead to incorrect inferences and conclusions and overlooks the dual character of semicontinuous data. For instance, Baldwin et al. [8] showed that a traditional linear multilevel model falsely indicated that there was no change in the semicontinuous outcome daily physical activity (PA) over the course of the study, not detecting that with time participants became less likely to engage in PA. An alternative approach to study semicontinuous data using traditional models is to exclude time-intervals with zeros, i.e. include only time-intervals in which dietary intake occurred to study how much but not whether dietary intake occurred. However, this causes loss of important information [9] and can cause bias in the parameter estimates [10, 11] (as outlined in the discussion). Furthermore, a common approach is to study whether but not how much dietary intake occurred through multilevel logistic regressions (e.g. [7, 12–14]). However, if the amounts of the consumed foods/drinks are also captured, available data with important implications are disregarded.

### Statistical approach for semicontinuous outcomes

A generally less known, but highly informative statistical approach for semicontinuous outcomes is two-part modelling. It treats zeros and nonzeros of the outcome separately as generated by a dual process. The zero part (occurrence indicator – e.g. has an individual eaten in a given time-interval?) and continuous/positive part (intensity indicator – e.g. if an individual ate in a given time-interval, how much was eaten?) of the outcome follow different distributions. Two-part models combine these two distributions: a logistic or probit regression for zeros (e.g. to predict whether an individual eats) and a linear or generalized linear regression for positive values (e.g. to predict how much is eaten, if an individual eats).

Two-part modelling assesses these two parts (e.g. the probability of eating and the amount that is eaten) while accounting for the potential dependency between them. The importance of taking this potential dependency into account was highlighted by Olsen and Schafer who were the first to extend these models to longitudinal data [15].

Hence, multilevel two-part modelling not only allows studying dietary intake as a dual process, but also overcomes the challenges of semicontinuous data. It does not overlook relevant information and provides additional and novel insights. It differentiates between factors either influencing the occurrence or the amount of dietary intake or both. If both, it can be assessed whether a factor’s importance differs across the two parts.

Even though the use of two-part models is less common in most research fields, it has become popular for example in the following fields: Medical costs [16, 17], substance use disorder [18–22] and PA [8, 23–25]. Two-part models have also been applied to nutritional data in order to estimate usual intake of episodically consumed foods [26]. However, to the best of our knowledge, multilevel two-part modelling has not yet been applied to studying dietary intake in daily life. Furthermore, most publications on multilevel two-part modelling used statistical software which is less common (e.g. WinBUGS [22]) or not free to use (e.g. SAS Proc NLMIXED [16], “gsem” command in Stata [8, 25]).

### Objective

This paper is the first to introduce multilevel two-part modelling as a novel analytical approach to study dietary intake in daily life. We believe that the importance of multilevel two-part models in behavioural nutrition as well as other behavioural research fields (e.g. PA) is growing. Practical guidance is needed to facilitate the implementation of these rather complex models, particularly in commonly used and freely available software. For this reason, this paper proposes a multilevel two-part model combining a multilevel logistic and a multilevel gamma regression to study dietary intake in daily life using R [27], one of the most commonly used data software programs which is freely available and therefore accessible to everyone. In the present work, we use the R-package brms [28, 29] which is based on Bayesian inference. We chose this package because it allows great flexibility in this specific model. Furthermore, its syntax is very similar to the syntax of other and likely more widely used multilevel packages in R (nlme [30]; lme4 [31]). This has the benefit that readers familiar with multilevel modelling in R can more easily build upon prior experience. We assume that readers have basic knowledge of multilevel modelling (e.g. multilevel structure of the data, random effects). Readers not familiar with these basic concepts are referred to introductory literature on multilevel modelling (e.g. [32, 33]). To ensure readers who are new to Bayesian statistics are able to follow, the basic concept of Bayesian inference is briefly introduced in Additional file 1a.

The aim of this paper is to introduce multilevel two-part modelling as a novel analytical approach to study dietary intake in daily life and provide easy-to-follow guidance on its practical application. To do so, the methods section covers (1) general model specifications of the proposed model, (2) a brief overview of brms and the general implementation of the proposed model in brms and (3) the description of the data used in this paper. The results section outlines the results of the exemplary analyses in detail, in order to provide practical guidance on the model specification and interpretation. Data and R code are provided in Additional files 2 and 3.

## Methods

### Multilevel two-part model for semicontinuous dietary data

In order to study dietary intake in daily life, we propose a multilevel two-part model which combines a multilevel logistic regression for zeros to predict whether an individual eats and a multilevel gamma regression for right-skewed positive values to predict how much is eaten, if an individual eats. Here, repeated assessments (Level 1) of the semicontinuous variable dietary intake are nested within individuals (Level 2). We chose the multilevel gamma regression for positive values as it does not require data transformation (e.g. logarithmizing) and beyond that performed well for right-skewed continuous PA data in Baldwin et al. [8]. A gamma distribution is a continuous probability distribution which is commonly used to model continuous variables which can only be positive and follow a skewed distribution.

In the following we briefly introduce the model specifications. A more comprehensive introduction to the model specifications can be found in Additional file 4.

The variable y_*ij*_ represents the semicontinuous dietary intake response from subject *j* (*j* = 1, …, *m*) at time point *i* (*i* = 1, …, *n*_*i*_). We are interested in two parts of this variable: (1) Did the participant eat? In other words, is y_*ij*_ = 0 or y_*ij*_ > 0 (illustrated in Fig. 1b)? (2) If the participant ate, how much was eaten? In other words, what is the expected value of y_*ij*_, if y_*ij*_ > 0 (illustrated in Fig. 1c)?

**Fig. 2:**
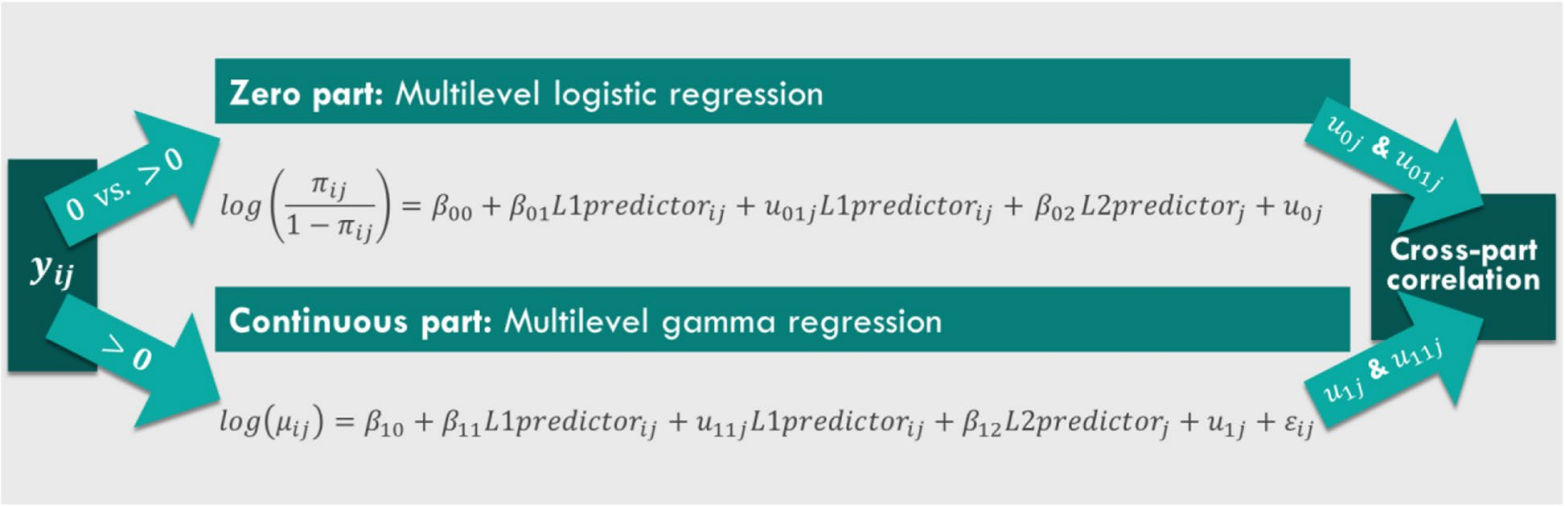
Illustration of the proposed multilevel two-part model

A multilevel logistic regression is used for part (1) of the semicontinuous variable. It predicts the log-odds of no eating for person *j* at time point *i* ($\mathrm{log}\left(\frac{\mathrm{\pi_{ij}}}{1-\mathrm{\pi_{ij}}}\right)$).^2^ Figure 2 shows that the log-odds of no eating can be predicted as a function of Level-1 and Level-2 covariates.

A multilevel gamma regression is used for part (2) of the semicontinuous variable. It predicts the expected log amount of dietary intake of person *j* at time point *i* (*log(μ*_*ij*_*)*) when eating occurred. *μ*_*ij*_ is modelled on the log scale due to the fact that the gamma distribution only supports positive values. However, it is important to highlight that this does not change the data as would log-transforming the data before running the model. The metric of the variable remains unchanged and the slope coefficients can be interpreted through exponentiation (demonstrated in the results). Figure 2 shows that a function of Level-1 and Level-2 covariates can be used to predict the (log) amount of dietary intake.

*L1predictor*_*ij*_ in Fig. 2 represents a Level-1 covariate assessed at time point *i* in person *j*, e.g. participant *j*’s momentary affect at measurement occasion *i*. *L2predictor*_*j*_ is a Level-2 covariate of person *j*, e.g. participant *j*’s BMI. *β*_*00*_ and *β*_*10*_ are the overall intercepts. The coefficients *β*_*01*_ and *β*_*11*_ represents the expected change for a one-unit increase in *L1predictor*. The expected change for a one-unit increase in *L2predictor* is expressed by *β*_*02*_ and *β*_*12*_. *u*_*0j*_ and *u*_*1j*_ represent the random intercepts of person *j*, i.e. person-specific deviation from the overall intercept. *u*_*01j*_ and *u*_*11j*_ denote the random effects of *L1predictor* in person *j*, i.e. person-specific differences in the effect of *L1predictor*. The error term ${\varepsilon }_{ij}$ in the continuous part of the model denotes the Level-1 residual, i.e. difference between the predicted value and the observed value of person *j* at time point *i*. The first subscript 0 or 1 of the parameters indicates that the equation refers to the zero or the continuous part of the model, respectively. Part specific interpretations of the parameters can be found in Table 1.

**Table 1:** Overview of the most relevant parameters and their interpretation

**Parameter**	**Description**	**Interpretation**
*Zero part*		
*β*_*00*_	overall intercept	mean of the log-odds of no eating across all participants when all predictors are equal to 0
*β*_*01*_	fixed effect of *L1predictor*	expected change in log-odds of no eating for a one-unit increase in *L1predictor*
*β*_*02*_	fixed effect of *L2predictor*	expected change in log-odds of no eating for a one-unit increase in *L2predictor*
*u*_*01j*_	random effect of *L1predictor* in person *j*	person-specific differences in the effect of *L1predictor* on the log-odds of no eating
*u*_*0j*_	random intercept of person *j*	person-specific differences in the log-odds of no eating
$\sqrt{{{\sigma }^{2}}_{{u}_{0}}}$	standard deviation of the random intercept *u*_*0*_	variation of between-person differences in the log-odds of no eating
$\sqrt{{{\sigma }^{2}}_{{u}_{01}}}$	standard deviation of the random effect *u*_*01*_	variation of between-person differences in the effect of *L1predictor* on the log-odds of no eating
*Continuous part*		
*β*_*10*_	overall intercept	mean of the (log) amount consumed across all participants when all predictors are equal to 0 given that dietary intake occurred
*β*_*11*_	fixed effect of *L1predictor*	expected change in the (log) amount consumed for a one-unit increase in *L1predictor*
*β*_*12*_	fixed effect of *L2predictor*	expected change in the (log) amount consumed for a one-unit increase in *L2predictor*
*u*_*11j*_	random effect of *L1predictor* in person *j*	person-specific differences in the effects of *L1predictor* on the (log) amount consumed
*u*_*1j*_	random intercept of person *j*	person-specific differences in the (log) amount consumed
$\sqrt{{{\sigma }^{2}}_{{u}_{1}}}$	standard deviation of the random intercept *u*_*1*_	variation of between-person differences in the expected (log) amount consumed
$\sqrt{{{\sigma }^{2}}_{{u}_{11}}}$	standard deviation of the random effect *u*_*11*_	variation of between-person differences in the effect of *L1predictor* on the expected (log) amount consumed
*Cross-part correlation*		
${\rho }_{{u}_{0}{u}_{1}}$	correlation between the random intercepts *u*_*0*_ and *u*_*1*_ of the zero and continuous part	correlation between the person-specific differences in the log-odds of no eating and the person-specific difference in the (log) amount consumed

The two processes modelled through the multilevel logistic and gamma regression are likely not independent. Therefore, an important consideration in two-part modelling, as highlighted by Olsen and Schafer [15] for longitudinal data, is whether an individual’s average probability of eating is related to the individual’s average amount consumed when the individual eats. In other words, the average proportion of occasions on which the participant does not eat may be related to the average (log) amount of dietary intake during eating occasions. To account for this potential relation, the correlation between the random effects across the two parts (e.g. ${\rho }_{{u}_{0}{u}_{1}}$), often called cross-part correlation, is modelled (illustrated in Fig. 2). The number of modelled correlations is determined by the number of random effects included in the model (see Additional file 4 for details).

An overview of the most relevant parameters in the proposed multilevel two-part model is provided in Table 1.

More general overviews of (multilevel) two-part models can be found in the following literature: Neelon et al. [34, 35] provide an overview as well as case studies on zero-modified count and semicontinuous data, marginally also covering longitudinal data. Liu et al. [36] discuss statistical analyses of semicontinuous data in the cross-sectional as well as longitudinal setting. Farewell et al. [37] provide a review on two-part and related regression models for longitudinal semicontinuous as well as longitudinal count data.

### Multilevel two-part modelling in brms

#### brms

The R-package brms [28, 29] supports Bayesian multilevel modelling and is implemented via the probabilistic programming language Stan [38]. For readers who are new to Bayesian statistics, a brief introduction is provided in Additional file 1a. We recommend Depaoli et al. [39] as well as van de Schoot and Depaoli [40] to readers who are interested in a broader introduction to Bayesian statistics in the context of health psychology.

We chose brms for this paper for a number of reasons: Firstly and most importantly, the major advantage of brms is that it uses a lme4-like formula syntax. lme4 is one of the most commonly used R-packages for multilevel modelling which will facilitate the initial familiarization with brms for those readers who are familiar with multilevel modelling in R. Secondly, it does not require any data preprocessing (e.g. dividing the semicontinuous outcome into two variables, a dichotomous and a continuous variable) as other software programs do (e.g. gsem in Stata). Thirdly, it offers great flexibility in the model specification (see [28, 29] for details).

### Multilevel two-part model in brms

The proposed multilevel two-part model combining a multilevel logistic and a multilevel gamma regression can be run in brms through the family *hurdle_gamma*.

The general syntax of the model looks as follows:
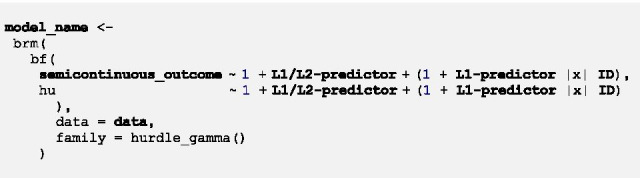


First of all, the name with which the fitted model will be saved in the R-Environment is specified. The *brm*-function indicates that a Bayesian generalized (non-)linear multilevel model is fitted. *bf* (short for *brmsformula*) is used for setting up the model formula. The upper part within *bf* represents the formula for the multilevel gamma regression for positive values.^3^ The bottom part indicated by *hu* shows the formula for the multilevel logistic regression for zero values. The two parts of the model are specified after ~ through a formula almost identical to lme4-syntax. The initial *1* represents the overall intercepts which are followed by Level-1 and/or Level-2 predictors with fixed effects*.* Within parentheses, random effects of Level-1 predictors can be specified after the random intercept *1. |x|* specifies random effects of the same participant to be correlated across the two parts of the model, i.e. cross-part correlations are modelled (denoted as the cross-part covariance matrix ***Σ***_***01***_ in Additional file 4). *x* within | | was chosen arbitrarily and can be exchanged for any letter or digit. After *|x|* the grouping variable is specified, in longitudinal data the variable indicating the participant ID. *data* indicates which data frame is used for the analysis. Bold parts of the syntax have to be customized.

Additional parameters can—and in some cases must—be specified within the *brm*-function to adapt the sampling algorithm (see Additional file 1a for a brief introduction to Bayesian sampling). brms runs 4 Markov chains with 2000 iterations each by default. The number of chains and iterations per chain can be customized through the arguments *chains* and *iter*. Unless otherwise specified through the argument *warmup*, half of the iterations are warm-up iterations (in the default setting: 2000/2 = 1000). If a model does not converge, brms provides a link to a website [41] with detailed information on recommended modifications (e.g. increase the number of iterations) to make the model converge. The argument *set_pior* can be used to incorporate prior information. However, due to a lack of prior information we exclusively use the default priors of brms in this paper which are very weakly informative and therefore influence the results as little as possible.

### Data and material

The following research question is assessed exemplary within this paper: “Do momentary energetic arousal and gender predict the occurrence of energy intake and/or the amount of energy consumed within time-intervals in which energy intake occurred in daily life?” This question was chosen purely for illustrative purposes. We do not test specific a prior hypotheses with these analyses.

Data were collected within the Eat2beNICE-APPetite-study. This study captures dietary intake and related factors through EMA using the APPetite-mobile-app (details on the APPetite-mobile-app can be found in Ruf et al. [45]). Dietary intake was captured in an event-contingent fashion and used to quantify energy and nutrient intake. Momentary energetic arousal was assessed signal-contingent through 8 semi-random prompts per day. Participants used the app for three consecutive days. Hence, energetic arousal was assessed at up to 24 time points.

**Fig. 3:**
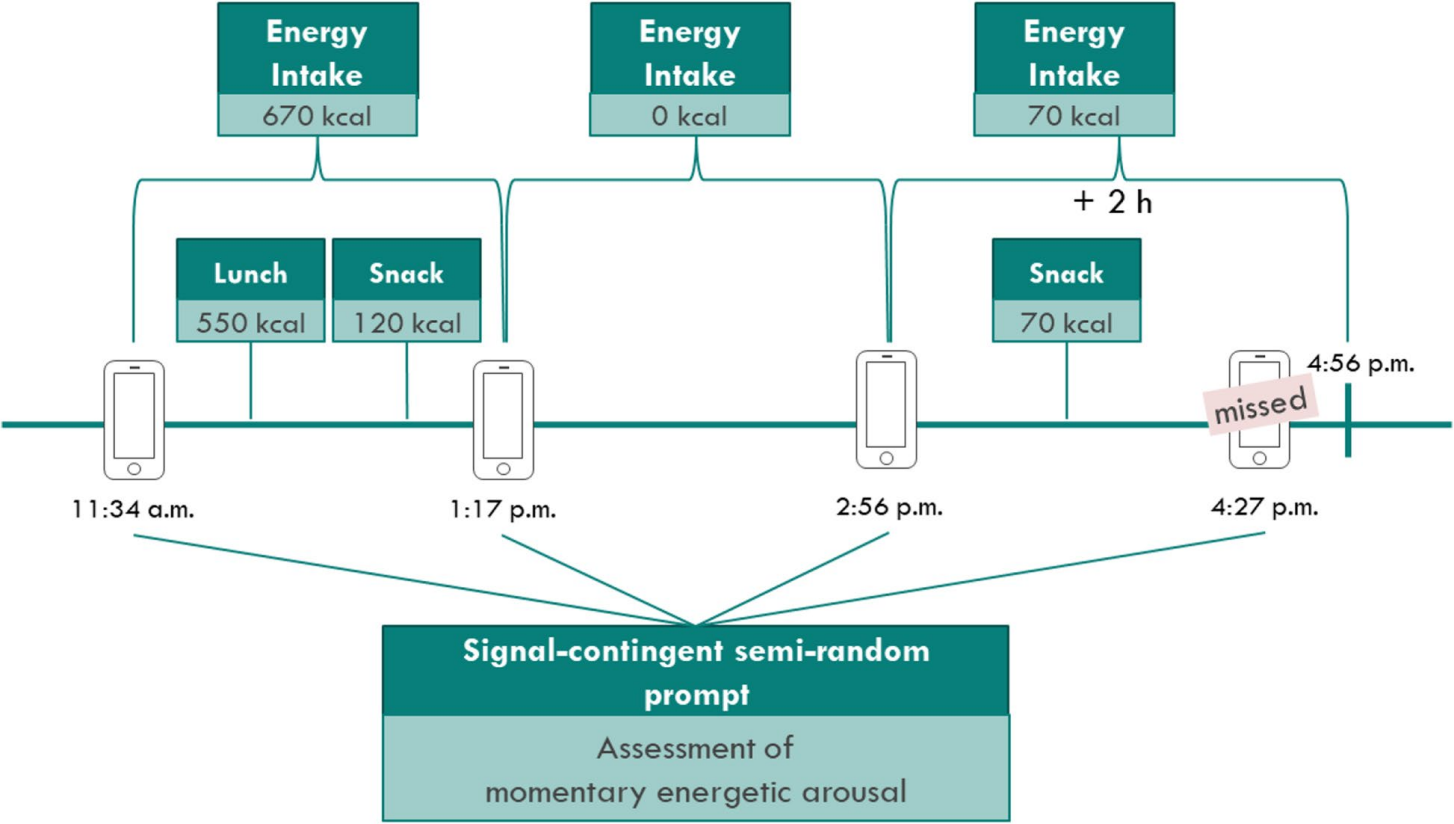
Illustration of the data pre-processing procedure

Each assessment of energetic arousal was matched to subsequent energy intake (in kcal). Subsequent energy intake was defined as the sum of any intake of energy within the time until the next assessment of energetic arousal or within the next 2 h if the time between two assessments was more than 2 h (e.g. because a prompt was missed) (see Fig. 3 for an illustration).

The dataset and the R code used in this paper can be found in Additional files 2 and 3. The dataset contains 2044 time points from 99 participants. 48.4% (989/2044) of time-intervals show no energy intake and are therefore equal to 0. The mean of non-zero values is 444.5 kcal. The dataset is in long-format (that is, repeated measurements for each participant are reported in separate lines of the dataset) and contains the variables shown in Table 2.

**Table 2:** Variable overview

**Name**	**Description**	**Coding**
ID	subject ID	1, 2, 3, …, 99
alarm	number of prompt (maximum = 24)	1, 2, 3, …, 24
day	day 1 to 3	1, 2, 3
time	numeric time of random alarm	e.g. 8.5 for 8:30 a.m
energy_intake	energy intake in kcal	
gender	participants’ gender	0 = male, 1 = female
EA	momentary energetic arousal, person-mean-centered	

Analyses were run using version 4.0.5 of R, version 1.4.1106 of RStudio (RStudio Inc., Boston, MA, USA [42]), version 2.15 of brms and version 2.21.2 of rstan [43].

## Results

### Intercept only model

**Fig. 4:**
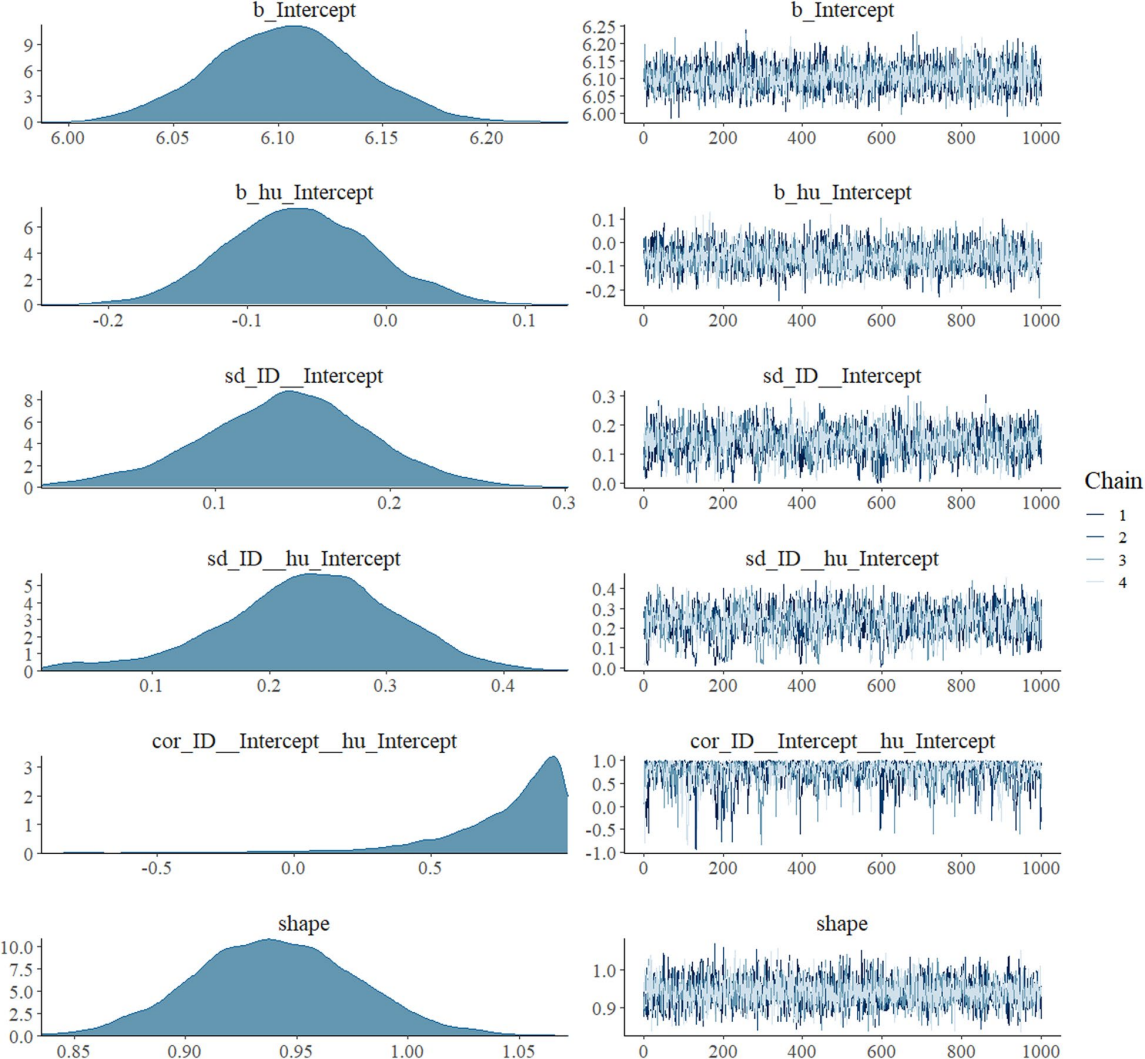
Density and trace plots of each model parameter of the intercept only model

First of all, we specify and run an intercept only model (also called empty model or null model). As the name implies, it does not contain any predictors, only intercepts. The model syntax looks as follows:
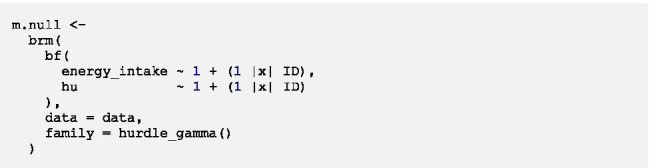


When running the model, the following code appears progressively in the console:
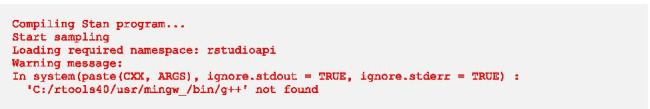


First, it shows that Stan is being compiled. A few moments later, sampling is started and the viewer opens. By refreshing the viewer, the progress of the sampling can be monitored. When the model is fitted, a warning is printed. However, this warning can be ignored as it does not affect the model estimation and will be removed in the next release of rstan [44]. As we do not get any other warnings, the model seems to have converged. However, to reassure the quality of the parameter estimates, additional information regarding the construction of the posterior distribution should be obtained. To check convergence, we have a look at density and trace plots of the parameter estimates. These plots can be produced by running the command *plot(m.null)* and are shown in Fig. 4.

Density plots of model parameters should be clearly unimodal which seems to be the case in this model. Trace plots show each sampled parameter estimate from the first to the 1000^th^ iteration of each of the four chains after warm-up. The estimates should circle around a single value to indicate convergence. The trace plots in Fig. 4 indicate convergence as the estimates hover around a single value. If the density and trace plots suggest that the model has not converged, the model should be run with more iterations. The potential scale reduction factor evaluates convergence through assessing differences between the chains (between-chain variance/within-chain variance) and should be close to 1. It is given for each parameter in the brms output in the column *Rhat* and is close to 1 if no warning is displayed. As the plots do not show any signs of nonconvergence and no relevant warnings are displayed, we can have a look at the model estimation. To do so, we run the command *summary(m.null)* and get the following results^4^:
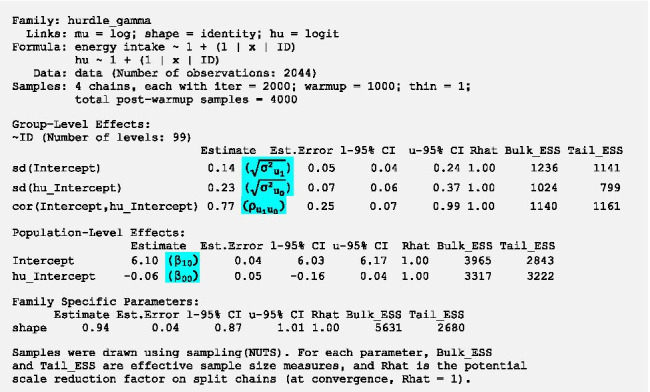


First of all, we double-check that the values in the column *Rhat* are close to 1. All Rhats are equal to 1.00 in this model, so the parameter estimates can be deemed trustworthy.

In the section *Population-Level Effects* which comprises fixed effects, we get two intercepts, one for the gamma part of the model *β*_*10*_ (= *Intercept*) and one for the logistic part *β*_*00*_ (= *hu_Intercept*). In brms, point estimates of parameters represent the mean of the respective posterior distribution. Estimates of the gamma part are modelled on the log scale as the outcome can only be positive. Hence, to obtain the estimate of the intercept in the original metric (kcal), we calculate the exponential of *β*_*10*_ (*exp(6.1)* = 445.9). This indicates that in time-intervals in which energy intake occurred we expect an individual to consume on average 445.9 kcal. This value should be close to the mean of non-zero values in the original data as the group mean is the best estimate in models without predictors. In our data the mean of positive values is 444.5 which is very close to the model estimate.

Estimates of the logistic part are modelled on the logit scale which accommodates the restricted range of probabilities (between 0 and 1). The intercept *β*_*00*_ represents the average log-odds of no energy intake across all participants. To transform the log-odds to the probability of no energy intake, we can use the inverse logit function in Eq. (1) or alternatively the *plogis*-function in R.

1 $$\pi = \frac{\mathrm{exp}({\beta }_{00})}{1+ \mathrm{exp}({\beta }_{00})}=\frac{\mathrm{exp}(-0.06)}{1+ \mathrm{exp}(-0.06)}=plogis\left(-0.06\right)=0.485$$

We get a mean probability of no energy intake of 0.485 (= 48.5%). We can check whether this estimate is reasonable through looking at the percentage of time-intervals without energy intake within the original data. In 48.4% (989/2044) of time-intervals energy intake is equal to zero which is close to the estimate of the intercept. We recommend always checking the model implied estimates from the intercept only model against the descriptive sample estimates to ascertain that the model was specified correctly and that the sample estimates could be reproduced with reasonable accuracy.

Now we have a look at the random effects (*Group-Level Effects* in brms). *Sd(Intercept)* ($\sqrt{{{\sigma }^{2}}_{{u}_{1}}}$) represents the variation of the random intercept *u*_*1*_ of the gamma part, i.e. person-specific variation in the mean of (log) energy intake. Mean (log) energy intake varies between participants with a *SD* of 0.14 (95%-credible interval [CI] 0.04–0.24). As effects are assumed to be multivariate normally distributed, we can calculate the range in which the mean energy intake of 95% of participants is located (Intercept ± 1.96 *SDs*). The mean energy intake of 95% of participants is between 338.86 (exp(6.1–0.14*1.96)) and 586.63 kcal (*exp(6.1* + 0.14*1.96)) in time-intervals in which energy intake occurred. Furthermore, participants differ in the mean log-odds of no energy intake with a *SD* of 0.23 (95%-CI 0.06–0.37) shown by the variation of the random intercept *u*_*0*_ of the logistic part *sd(hu_Intercept)* ($\sqrt{{{\sigma }^{2}}_{{u}_{0}}}$). For 95% of participants the probability of no energy intake is between 0.38 (plogis(-0.06–0.23*1.96)) and 0.6 (*plogis(-0.06* + 0.23*1.96)).

The fairly strong positive cross-part correlation between the random intercepts (${\rho }_{{u}_{0}{u}_{1}}$) of 0.77 indicates that participants who consume on average more energy within time-intervals in which energy intake occurs have on average a higher probability of no energy intake.

### Random intercept model with Level-2 predictor

Now we want to include a fixed effect of the Level-2 predictor gender in both parts of the model by running the following code:
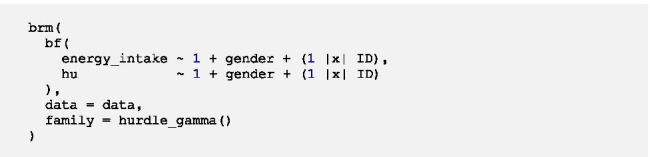


We do not get any warnings regarding nonconvergence and the density and trace plots do not indicate convergence problems, therefore we can interpret the model estimates^5^:
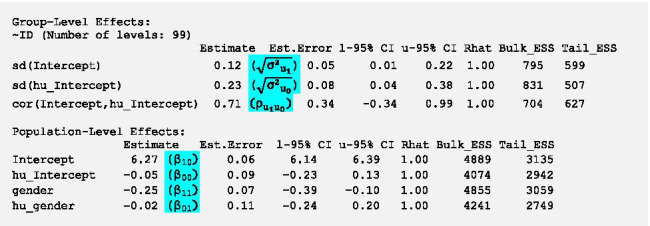


In this model the intercept *β*_*10*_ of the gamma part of the model represents the mean log energy intake for men (*gender* = 0). Male participants consume on average 528.48 kcal (*exp(β*_*10*_*)* = *exp(6.27)*) in time-intervals in which energy intake occurred. Results show that gender has a fixed effect on the mean log energy intake in time-intervals in which energy intake occurs as the 95%-CI of *β*_*11*_ does not include 0. To interpret the regression coefficient of the fixed effect of gender, we can get the rate decrease in energy intake associated with a one-unit increase in *gender* through exponentiation of *β*_*11*_. Hence, women (*gender* = 1) consume on average around 22% less energy (*exp(β*_*11*_*)* = *exp(-0.25)* = *0.78*) in time-intervals in which energy intake occurred compared to men. However, women and men do not differ in the probability of no energy intake as the fixed effect of gender is not relevant for the prediction in the logistic part of the model (95%-CI of *β*_*01*_ includes 0).

We get three estimates within the random effects. There is between-person variation in the log energy intake ($\sqrt{{{\sigma }^{2}}_{{u}_{1}}}$=0.12, 95%-CI 0.01–0.22) in time-intervals in which energy intake occurred as well as in the log-odds of no energy intake ($\sqrt{{{\sigma }^{2}}_{{u}_{0}}}$=0.23, 95%-CI 0.04–0.38). The cross-part correlation ${\rho }_{{u}_{0}{u}_{1}}$ is still fairly strong (0.71), suggesting that participants who consume on average more energy in time-intervals in which energy intake occurs have on average a higher probability of no energy intake. However, what we see here is that the 95%-CI of ${\rho }_{{u}_{0}{u}_{1}}$ includes 0 and is wider compared to the intercept only model indicating that the estimation is rather inaccurate (95%-CI -0.34–0.99).

### Random slope model with Level-1 predictor

Now we want to include the Level-1 predictor momentary energetic arousal in both parts of the model as fixed and random effects. As there was no fixed effect of gender in the logistic part of the model, we only include gender in the gamma part. To do so, we fit the following model:
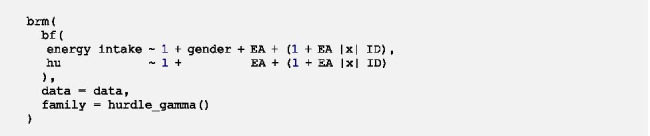


We do not get any warnings regarding nonconvergence and the density and trace plots do not indicate serious convergence problems, therefore we can interpret the model estimates:
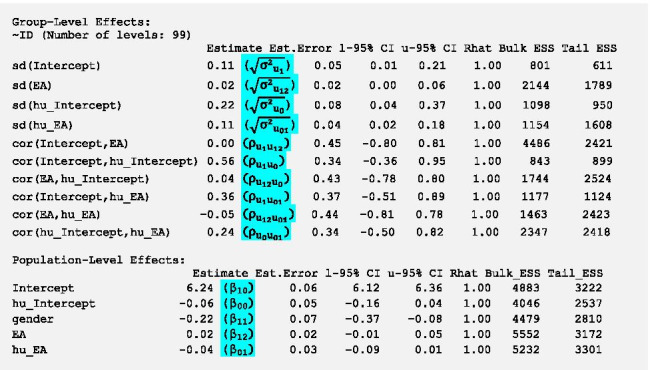


Again we see the meaningful fixed effect of gender in the gamma part (*β*_*11*_). However, there is no fixed effect of energetic arousal in either of the two parts (95%-CI include 0). That is, there is no evidence that participants were more likely not to eat when their energetic arousal was higher than usual, *β*_*01*_ = -0.04 (95%-CI -0.09–0.01). There was also no evidence that participants consumed more energy when their energetic arousal was higher than usual, *β*_*12*_ = 0.02 (95%-CI -0.01–0.05). Notice, however, that the random effect for energetic arousal in the logistic part suggests that the effect of energetic arousal on the log-odds of no energy intake varies across participants with a *SD* of 0.11 (95%-CI 0.02–0.18). Hence, for 95% of participants the effect of energetic arousal on the log-odds of no energy intake is between -0.26 (-0.04–0.11*1.96) and 0.18 (-0.04 + 0.11*1.96). This suggests that on average there is no association of energetic arousal with the probability not to eat. However, for some participants, higher arousal may be associated with a higher probability not to eat. Whereas for others, higher arousal may be associated with a lower probability not to eat. The random effect for energetic arousal in the gamma part was smaller and the lower bound of the 95%-CI was 0.00. Note that non-positive estimates for *SD* are not permitted, and the lower bound of the CI for this parameter will therefore always be positive. This suggests that inter-individual differences in the effect of energetic arousal on the amount of energy intake are small and possibly not statistically meaningful.

We get ten estimates within the random effects: 4 *SD*s and 6 correlations (as shown in expression (6) in Additional file 4). We see that the cross-part correlation ${\rho }_{{u}_{0}{u}_{1}}$ between the random intercepts is weaker than in the previous models (0.56) and that the 95%-CI of all correlations is very wide indicating that it is not possible to get accurate estimates (see also the platykurtic posterior distributions in Additional file 5).

## Discussion

Studying dietary intake through multilevel two-part modelling is a methodologically as well as conceptually promising approach. It accounts for the semicontinuous data structure and offers novel and distinct insights in terms of the occurrence as well as the amount of dietary intake. Results of this paper highlight that the differentiation between the two processes reveals process-specific associations which cannot be detected through traditional multilevel modelling. For instance, we found that gender is associated with the amount consumed during eating occasions, but not with the probability of eating. The model we propose overcomes a number of limitations of traditional modelling when analysing semicontinuous data: (1) accounts for the zero-inflation by introducing two model parts, a zero and a continuous part, which avoids incorrect inferences (as shown by Baldwin et al. [8]), (2) accommodates the skewness of the continuous part of the outcome by applying a gamma regression which does not rely on controversial transformation of the outcome and does not change the metric of the data, and (3) considers the dependency between the two model parts by modelling the cross-part correlation which prevents bias in parameter estimation as would running separate models (as outlined below). Despite its potential, multilevel two-part modelling is still missing in the statistical repertoire of most researchers. This may be due to the fact that these models are rather complex and therefore require initial training. However, we believe that multilevel two-part models are the most appropriate and valid method to study semicontinuous outcomes and therefore are worth the training. To facilitate the initial training and encourage other researchers to use these models, this paper offers an application-oriented introduction to multilevel two-part modelling.

The R-package brms used in this paper offers a user-friendly and freely available option for fitting multilevel two-part models. It is particularly intuitive for users familiar with lme4 and Bayesian statistics (see Additional file 1b for a brief overview of similarities and differences between Bayesian and frequentist-based two-part models). We believe that multilevel two-part models are of particular interest to those researchers who are familiar with traditional multilevel modelling.

For demonstration and simplicity purposes, we have focused on multilevel two-part models with fixed and random effects. However, extensions to the model (e.g. cross-level-interactions) are straightforward.

We found fairly strong to moderate positive cross-part correlations (0.77, 0.71, 0.56) indicating that participants who consume on average more energy during eating occasions have on average a higher probability not to eat. However, we have faced some estimation inaccuracies of the cross-part correlations: the more predictors we included in the model, the wider the 95%-CIs got. Nonetheless, we do not recommend fitting separate models as ignoring the cross-part correlation can induce bias in regression coefficients as well as variance components [10, 11]. Not accounting for the cross-part correlation can cause bias particularly in the continuous part of the model. This can be explained by the fact that the zero part determines the cluster size of the continuous part of the model (e.g. the number of observations with dietary intake within an individual). For instance, we found moderate to strong cross-part correlations. Hence, an individual less likely to eat will have fewer observations in the continuous part of the model but the few observations will contain larger amounts. An individual eating more frequently will have more observations in the continuous part which will contain smaller amounts. As a result, higher values of dietary intake will be underrepresented and smaller values will be overrepresented. Su et al. [10] outline that even when researchers are only interested in the continuous part of the semicontinuous outcome and therefore chose to fit a single model, the described bias will still be present.

To run the proposed multilevel two-part model, data on dietary intake as well as individual and/or situational factors have to be collected. Dietary intake can either be captured event- (i.e. when food is consumed [45]), signal- (e.g. time since the last prompt) or time-contingently (e.g. within the last hour [1]), while individual and/or situational factors have to be assessed either signal- or time-contingently. The proposed model cannot be applied to simple event-contingent sampling protocols (e.g. dietary intake and factors of interest assessed only when food is consumed).

While first empirical evidence [1] as well as results of this paper support the importance of distinguishing between the occurrence of eating and the amount that is eaten, future research is needed to verify the conceptual relevance of studying dietary intake as a dual process. We believe that multilevel two-part models will contribute to a better understanding of which situational and individual factors are associated with an increased probability of eating and/or with an increased amount of dietary intake. Findings in this area offer new perspectives and enable the development of tailored interventional strategies. For instance, in the context of preventing and treating overweight and obesity two types of interventions are needed: (1) interventions customized to reduce the probability of dietary intake and therefore reduce the number of eating occasions within a day, (2) interventions tailored to reduce the amount eaten within eating occasions to prevent overeating.

In this paper, we applied multilevel two-part modelling to study factors influencing energy intake. However, multilevel two-part models can also be employed to study macro-nutrient intakes which are also semicontinuous in the Eat2beNICE-APPetite data. Furthermore, multilevel two-part modelling can also be applied to studies which capture food categories (e.g. vegetable intake), provided that the consumed amounts are also assessed. Findings in the context of macro-nutrient intake and food categories can be translated to the promotion of healthy eating, e.g. reducing the occurrence of sugar intake or boosting vegetable consumption within eating occasions. Hence, there are numerous ways multilevel two-part modelling can be applied in the context of studying dietary intake in daily life.

Beyond that, the model proposed in this paper can also be applied to other research contexts in which a semicontinuous outcome is present, including PA data in which zeros are a common problem [46] (e.g. daily PA data [8] or PA data in EMA studies [25]). In fact, almost all behavioural outcomes are likely to show semicontinuous characteristics which can be traced back to dual processes: one process determining whether the behaviour is shown and the other determining how long/intensive/often the behaviour is shown, e.g. smoking behaviour (Has an individual smoked? If so, how many cigarettes have been smoked?), social interaction (Has an individual engaged in social interaction? If so, how many minutes did the individual interact socially?) and purchase behaviour (Has an individual purchased anything? If so, how much money was spent?)—to name only a few. The shorter time-intervals are in which a specific behaviour is studied (e.g. daily diary and EMA studies), the more likely it is that the outcome is zero-inflated, i.e. the behaviour of interest is not shown. Therefore, as the number of these studies is continuously growing, so will the need for multilevel two-part modelling to study predictors of specific behaviours. This paper addresses this need by providing guidance on the implementation and interpretation of these rather complex models.

## Conclusions

To the best of our knowledge, this paper is the first to introduce multilevel two-part modelling as a novel analytical approach to study dietary intake in daily life. Distinguishing between factors influencing whether and how much is eaten is conceptually promising and offers new opportunities, particularly for customized nutritional interventions either targeting the occurrence of intake or the amount consumed during eating occasions. As we believe that the importance of EMA studies assessing factors influencing dietary intake in daily life is growing within the next years, this paper will help to establish an appropriate data analysis procedure that accounts for the dual character of dietary intake and the semicontinuous data structure.

## Supplementary Information

**Additional file 1.** Bayesian Statistics. **a.** Brief introduction to Bayesian statistics **b.** Similarities and differences between Bayesian and frequentist-based two-part models.

**Additional file 2.** Dataset.

**Additional file 3.** R Code.

**Additional file 4.** Model specifications of the proposed multilevel two-part model.

**Additional file 5:** Density and Trace Plots. **Figure 1.** Density and Trace Plots of the Random Intercept Model with Level-2 predictor *gender*. **Figure 2.** Density and Trace Plots of the Random Slope Model with Level-1 predictor energetic arousal (*EA*).

**Additional file 6:** Model Summaries. **Figure 1.** Model summary of the Random Intercept Model with Level-2 predictor *gender*. **Figure 2.** Model summary of the Random Slope Model with Level-1 predictor energetic arousal (*EA*).

## Data Availability

All data generated or analysed during this study are included in this published article and its supplementary information files.

## Abbreviations

EMA: Ecological momentary assessment
kcal: Kilocalories
PA: Physical activity
*SD*: Standard deviation
CI: Credible interval

## Footnotes

1 It is important to note that a normal distribution of the dependent variable itself is not an assumption of linear multilevel models. Instead, the residuals which reflect the unexplained part of the dependent variable have to follow a normal distribution. However, in practice the distribution of the residuals typically looks similar to the distribution of the dependent variable.

2 Note that the multilevel logistic regression predicts NO dietary intake (i.e. *y*_*ij*_ = 0). Typically logistic regressions predict *y* = 1. *π*_*ij*_ represents the probability not to eat for person *j* at time point *i*.

3 Note that the upper formula predicts only positive values even though the variable *semicontinuous_outcome* contains all values of the semicontinuous outcome, including zeros.

4 Note that the notations marked in blue are inserted by us for illustrative reasons.

5 To keep this paper short, we do not show any further density and trace plots and only show the relevant parts of the brms output (Population-Level and Group-Level effects). However, density and trace plots can be found in Additional file 5 and complete model summaries in Additional file 6.
