# Supplementary material for: Studying dietary intake in daily life through multilevel two-part modelling: a novel analytical approach and its practical application
Source: Int J Behav Nutr Phys Act. 2021 Sep 27;18:130. doi: 10.1186/s12966-021-01187-8 (PMC8477527; doi:10.1186/s12966-021-01187-8)
Supplement: Supplementary file 1 — Additional file 1. Bayesian Statistics. a. Brief introduction to Bayesian statistics b. Similarities and differences between Bayesian and frequentist-based two-part models. [file 12966_2021_1187_MOESM1_ESM.pdf]

Additional file 1. Bayesian statistics

a. Brief introduction to Bayesian statistics

Contrary to frequentist methods in which population parameter estimates are based on the sample data only, Bayesian statistics allow accounting for prior information. Based on the sample data as well as provided information on the prior distribution, a posterior distribution of each parameter is computed. However, the Bayesian approach can be used even if there is no reliable prior information available. In this case, instead of setting informative priors, noninformative priors are set which impact the posterior distribution as little as possible. Information from the posterior distribution can be summarized in the form of a point estimate of the respective parameter (the mean, median or mode of the posterior distribution) as well as a 95% credible interval (CI; 2.5 and 97.5 percentiles). The CI can be used to assess whether a regression coefficient is likely to be non-zero and hence relevant for the prediction of the outcome. If the CI does not include zero, it is reasonable to assume that the regression coefficient is different from zero (i.e. statistically significant). Note, however, that any type of CI could be used to determine whether or not the parameter is different from zero (this is similar to frequentist approaches in which the $p < 0.05$ criterion is ultimately arbitrary as well).

In contrast to frequentist methods, Bayesian estimation of the model proposed in this paper relies on Markov Chain Monte Carlo (MCMC) sampling, an approach that is based on simulation methods (see [1] for an overview on MCMC). It is necessary to understand the basic concept of MCMC to evaluate whether the model estimation has worked and the parameter estimates are reliable. MCMC combines the prior distribution and the information from the actual data through an iterative process obtaining a posterior distribution. Within this process, parameter values are sampled and used to update the posterior distribution. This procedure of sampling and updating is repeated many times (specified through the number of

iterations) and for multiple runs (specified through the number of chains). The first iterations of each chain are discarded and not used for inference, in order to reduce the influence of the starting values. These initial discarded iterations are often referred to the warm-up. To check whether the estimation process for each parameter estimate has converged, density and trace plots can be inspected (presented within the results). Another important criterion is the potential scale reduction factor which evaluates convergence through assessing differences between the chains. It is calculated for each parameter (between-chain variance/within-chain variance) and should be close to 1 (see [2–4] for details).

b. Similarities and differences between Bayesian and frequentist-based two-part models

The model we propose is based on Bayesian inference which raises the question how similar or different Bayesian two-part models are compared to frequentist-based models. Bayesian multilevel two-part models with non-informative priors, as used in this paper, are generally expected to yield similar estimates as maximum likelihood based approaches in the traditional frequentist framework [5]. However, it remains unclear whether or in which cases estimates of the Bayesian approach proposed in this paper are comparable to likelihood based estimates. Systematic simulation studies are needed to answer this question conclusively. Nonetheless, there are two distinct advantages of the Bayesian approach over the frequentist approach which should be taken into consideration: (1) Confidence intervals in the frequentist approach are often based on normality assumptions and are therefore defined as symmetrical (point estimate +/- 1.96 SE). As a result confidence intervals of random effects in the frequentist approach can include negative values. However, random effects are expressed by measures of variability (variance or SD) which cannot be negative. The Bayesian approach avoids this by incorporating prior distributions with only non-negative values which prevent negative estimates for random effect variances in the posterior distribution. (2) Maximum likelihood estimation can cause computational challenges and convergence difficulties, particularly

when models include complex random effect structures [5]. In general, Bayesian approaches have been shown to be more computational efficient [5,6] and are therefore suited particularly well for more complex data [5]. Nevertheless, future research is needed to examine if and when Bayesian approaches are more efficient than frequentist approaches for the two-part models used in this work.

1. van Ravenzwaaij D, Cassey P, Brown SD. A simple introduction to Markov Chain Monte–Carlo sampling. Psychon Bull Rev. 2018;25:143–54.

2. Gelman A, Rubin DB. Inference from Iterative Simulation Using Multiple Sequences. Stat Sci. 1992;7:457–72.

3. Brooks SP, Gelman A. General Methods for Monitoring Convergence of Iterative Simulations. J Comput Graph Stat. 1998;7:434–55.

4. Gelman A, Carlin JB, Stern HS, Dunson DB, Vehtari A, Rubin DB. Bayesian Data Analysis. 3rd ed. Chapman and Hall/CRC; 2013.

5. Smith VA, Neelon B, Preisser JS, Maciejewski ML. A marginalized two-part model for longitudinal semicontinuous data. Stat Methods Med Res. 2015;26:1949–68.

6. Ghosh P, Albert PS. A Bayesian analysis for longitudinal semicontinuous data with an application to an acupuncture clinical trial. Comput Stat Data Anal. 2009;53:699–706.
