# Supplementary material for: Studying dietary intake in daily life through multilevel two-part modelling: a novel analytical approach and its practical application
Source: Int J Behav Nutr Phys Act. 2021 Sep 27;18:130. doi: 10.1186/s12966-021-01187-8 (PMC8477527; doi:10.1186/s12966-021-01187-8)
Supplement: Supplementary file 4 — Additional file 4. Model specifications of the proposed multilevel two-part model. [file 12966_2021_1187_MOESM4_ESM.pdf]

Additional file 4. Model specifications of the proposed multilevel two-part model

In the following we use the notation from Baldwin et al. [1] to introduce the model specifications. However, we extend the model by introducing random effects in both parts of the model.

The variable y_{ij} represents the semicontinuous dietary intake response from subject *j* (*j* = 1, …, *m*) at time point *i* (*i* = 1, …, n_i). We are interested in two parts of this variable: (1) Did the participant eat? In other words, is $y_{ij}=0$ or $y_{ij}>0$? (2) If the participant ate, how much was eaten? In other words, what is the expected value of y_{ij}, if $y_{ij}>0$? To approach these questions, the semicontinuous variable y_{ij} is split into two parts:

$$y_{ij} \sim \begin{cases} \pi_{ij} & if\ y_{ij} = 0 \\ (1 - \pi_{ij})h(y_{ij}) & if\ y_{ij} > 0. \end{cases} \quad (1)$$

The upper part of expression (1) shows the probability π_{ij} for person *j* not to eat at time point *i*.[1] The bottom part represents the conditional probability distribution $h(y_{ij})$ for positive values, i.e. the expected amount eaten by person *j* at time point *i* if eating occurred. $h(y_{ij})$ is weighted by the probability that a person did eat which translates to subtracting the probability of no eating from 1 ($1 - \pi_{ij}$). A gamma distribution is used for $h(y_{ij})$.[2]

In the zero part of the model a multilevel logistic regression predicts the log-odds of no eating for person *j* at time point *i* ($\log\left(\frac{\pi_{ij}}{1-\pi_{ij}}\right)$).[3] Equation (2) shows that the log-odds of no eating can be predicted as a function of Level-1 and Level-2 covariates:

$$log\left(\frac{\pi_{ij}}{1-\pi_{ij}}\right) = \beta_{00} + \beta_{01}L1predictor_{ij} + u_{01j}L1predictor_{ij} + \beta_{02}L2predictor_j + u_{0j} \quad (2)$$

[1] Note that the multilevel logistic regression predicts NO dietary intake (i.e. $y_{ij}=0$). Typically logistic regressions predict $y=1$.

[2] The gamma distribution is assumed for Level-1 residuals (i.e. the part of the dependent variable which is not explained by the predictors of the model).

[3] Technically, the probability π_{ij} from expression (1) is not directly predicted, instead the log-odds of no eating are predicted, as the multilevel logistic regression is modelled on the logit scale to accommodate the restricted range of probabilities (between 0 and 1).

L1predictor$_{ij}$ in equation (2) represents a Level-1 covariate assessed at time point *i* in person *j*, e.g. participant *j*'s momentary affect at measurement occasion *i*. *L2predictor*$_j$ is a Level-2 covariate of person *j*, e.g. participant *j*'s BMI. β_{00} is the overall intercept, i.e. the mean of the log-odds of no eating across all participants when all predictors are equal to 0. The coefficient β_{01} represents the expected change in log-odds of no eating for a one-unit increase in *L1predictor*. The expected change in log-odds of no eating for a one-unit increase in *L2predictor* is expressed by β_{02}. u_{0j} represents the random intercept of person *j*, i.e. person-specific differences in the log-odds of no eating. u_{01j} denotes the random effect of *L1predictor* in person *j*, i.e. person-specific differences in the effect of *L1predictor* on the log-odds. The first subscript 0 of the parameters indicates that the equation refers to the zero part of the model (i.e. the logistic regression predicting if the participant did not eat).

Turning to the continuous part of the semicontinuous variable (see lower part of Equation 1), a multilevel gamma regression is used to predict the expected log amount of dietary intake of person *j* at time point *i* ($log(\mu_{ij})$) when eating occurred. μ_{ij} is modelled on the log scale due to the fact that the gamma distribution only supports positive values.

Equation (3) shows that a function of Level-1 and Level-2 covariates can be used to predict the (log) amount of dietary intake:

$$log(\mu_{ij}) = \beta_{10} + \beta_{11}L1predictor_{ij} + u_{11j}L1predictor_{ij} + \beta_{12}L2predictor_j + u_{1j} + \varepsilon_{ij} \quad (3)$$

L1predictor$_{ij}$ represents a Level-1 covariate of person *j* at time point *i*, *L2predictor*$_j$ a Level-2 covariate of person *j* and β_{10} the overall intercept, i.e. conditional mean of the (log) amount consumed across all participants when all predictors are equal to 0 given that the response is non-zero (i.e. dietary intake occurred). The regression coefficients β_{11} and β_{12} represent the expected change in the (log) amount consumed for a one-unit increase in *L1predictor* and *L2predictor*, respectively. The parameter u_{1j} reflects the random intercept of person *j*, i.e. person-specific differences in the expected (log) amount consumed. u_{11j} is the random effect

of predictor *L1predictor* in person *j*, i.e. person-specific difference in the effect of *L1predictor* on the expected (log) amount consumed. The error term ε_{ij} denotes the Level-1 residual, i.e. difference between the predicted value and the observed value of person *j* at time point *i*. The first subscript 1 of the parameters denotes the positive part of the model.

The two processes modelled through the multilevel logistic and gamma regression are likely not independent. Therefore, an important consideration in two-part modelling, as highlighted by Olsen and Schafer [2] for longitudinal data, is to account for this potential relation. To do so, the correlation between the random effects across the two parts (often called cross-part correlation) is modelled. Random effects are assumed to be jointly normal and possibly correlated as illustrated in expression (4). The random effects of the logistic part (summarized in the vector $\boldsymbol{u_{0j}}$) and of the gamma part (summarized in the vector $\boldsymbol{u_{1j}}$) are assumed to come from a multivariate normal distribution with a mean vector of 0 and an unknown covariance matrix $\boldsymbol{\Sigma}$:

$$\begin{bmatrix} \mathbf{u_{0j}} \\ \mathbf{u_{1j}} \end{bmatrix} \sim \mathrm{MVN}(\mathbf{0}, \boldsymbol{\Sigma}), \quad \boldsymbol{\Sigma} = \begin{bmatrix} \boldsymbol{\Sigma_0} & \\ \boldsymbol{\Sigma_{01}} & \boldsymbol{\Sigma_1} \end{bmatrix} \tag{4}$$

$\boldsymbol{\Sigma_0}$ and $\boldsymbol{\Sigma_1}$ are variance-covariance matrices of the random effects within the logistic and the gamma part of the model, respectively. $\boldsymbol{\Sigma_{01}}$ denotes the covariance matrix of the random effects across the two model parts, i.e. cross-part covariance matrix. The size of $\boldsymbol{\Sigma_0}, \boldsymbol{\Sigma_1}$ and $\boldsymbol{\Sigma_{01}}$ is determined by the number of random effects included in the model. Expression (5) shows exemplary the random effect variance-covariance matrix of a multilevel two-part model with the random intercept vectors $\boldsymbol{u_{0j}}$ and $\boldsymbol{u_{1j}}$.

$$\begin{bmatrix} \mathbf{u_{0j}} \\ \mathbf{u_{1j}} \end{bmatrix} \sim \mathrm{MVN}(\mathbf{0}, \boldsymbol{\Sigma}), \quad \boldsymbol{\Sigma} = \begin{bmatrix} \sigma^2{}_{u_0} & \\ \sigma_{u_0 u_1} & \sigma^2{}_{u_1} \end{bmatrix} \tag{5}$$

$\boldsymbol{\Sigma}$ contains $\sigma^2{}_{u_0}$, the person-to-person variability in the log-odds of no eating, $\sigma^2{}_{u_1}$, the person-to-person variability in the expected (log) amount consumed, and $\sigma_{u_0 u_1}$, the

covariance between these two random intercepts. Hence, three parameters (2 variances, 1 covariance) are estimated.

Expression (6) shows the random effects variance-covariance matrix of a multilevel two-part model with the random intercept vectors $\boldsymbol{u_{0j}}$ and $\boldsymbol{u_{1j}}$ as well as one random effect in the logistic (summarized in the vector $\boldsymbol{u_{01j}}$) and one in the gamma (summarized in the vector $\boldsymbol{u_{11j}}$) part of the model:

$$\begin{bmatrix} \mathbf{u_{0j}} \\ \mathbf{u_{1j}} \\ \mathbf{u_{01j}} \\ \mathbf{u_{11j}} \end{bmatrix} \sim \mathrm{MVN}(\mathbf{0}, \boldsymbol{\Sigma}), \quad \boldsymbol{\Sigma} = \begin{bmatrix} {\sigma^2}_{u_0} & & & \\ \sigma_{u_0 u_1} & {\sigma^2}_{u_1} & & \\ \sigma_{u_0 u_{01}} & \sigma_{u_1 u_{01}} & {\sigma^2}_{u_{01}} & \\ \sigma_{u_0 u_{11}} & \sigma_{u_1 u_{11}} & \sigma_{u_{01} u_{11}} & {\sigma^2}_{u_{11}} \end{bmatrix} \tag{6}$$

In this case, ten parameters (4 variances, 6 covariances) are estimated. ${\sigma^2}_{u_0}$, ${\sigma^2}_{u_{01}}$ and $\sigma_{u_0 u_{01}}$ are elements of the matrix $\boldsymbol{\Sigma_0}$, ${\sigma^2}_{u_1}$, ${\sigma^2}_{u_{11}}$ and $\sigma_{u_1 u_{11}}$ of $\boldsymbol{\Sigma_1}$ and $\sigma_{u_0 u_1}$, $\sigma_{u_0 u_{11}}$, $\sigma_{u_1 u_{01}}$ and $\sigma_{u_{01} u_{11}}$ of $\boldsymbol{\Sigma_{01}}$. Note that brms provides standard deviations and correlations instead of variances and covariances.

1. Baldwin SA, Fellingham GW, Baldwin AS. Statistical models for multilevel skewed physical activity data in health research and behavioral medicine. Heal Psychol. 2016;35:552–62.

2. Olsen MK, Schafer JL. A Two-Part Random-Effects Model for Semicontinuous Longitudinal Data. J Am Stat Assoc. 2001;96:730–45.
