# Supplementary material for: Studying dietary intake in daily life through multilevel two-part modelling: a novel analytical approach and its practical application
Source: Int J Behav Nutr Phys Act. 2021 Sep 27;18:130. doi: 10.1186/s12966-021-01187-8 (PMC8477527; doi:10.1186/s12966-021-01187-8)
Supplement: Supplementary file 5 — Additional file 5: Density and Trace Plots. Figure 1. Density and Trace Plots of the Random Intercept Model with Level-2 predictor gender. Figure 2. Density and Trace Plots of the Random Slope Model with Level-1 predictor energetic arousal (EA). [file 12966_2021_1187_MOESM5_ESM.pdf]

Additional file 5. Density and Trace Plots

Figure 1. Density and Trace Plots of the Random Intercept Model with Level-2 predictor *gender*

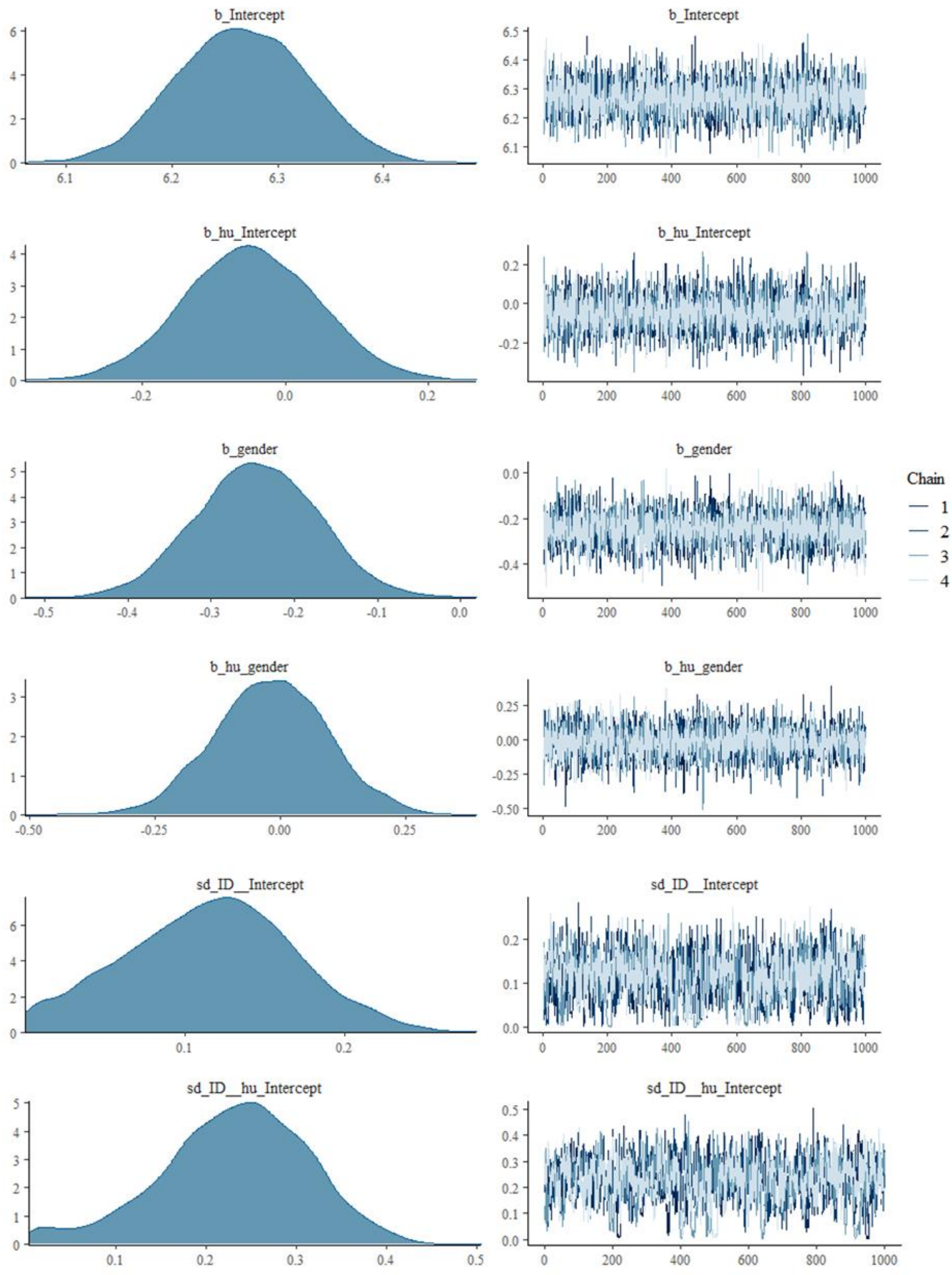

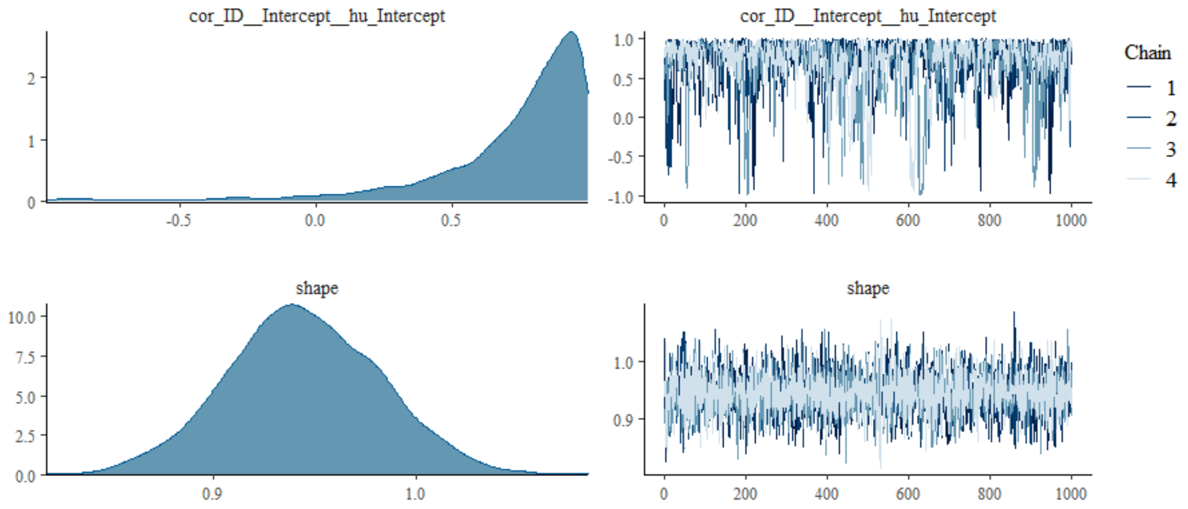
cor_ID__Intercept__hu_Intercept
cor_ID__Intercept__hu_Intercept
Chain
1
2
3
4
shape
shape

Figure 2. Density and Trace Plots of the Random Slope Model with Level-1 predictor energetic arousal (*EA*)

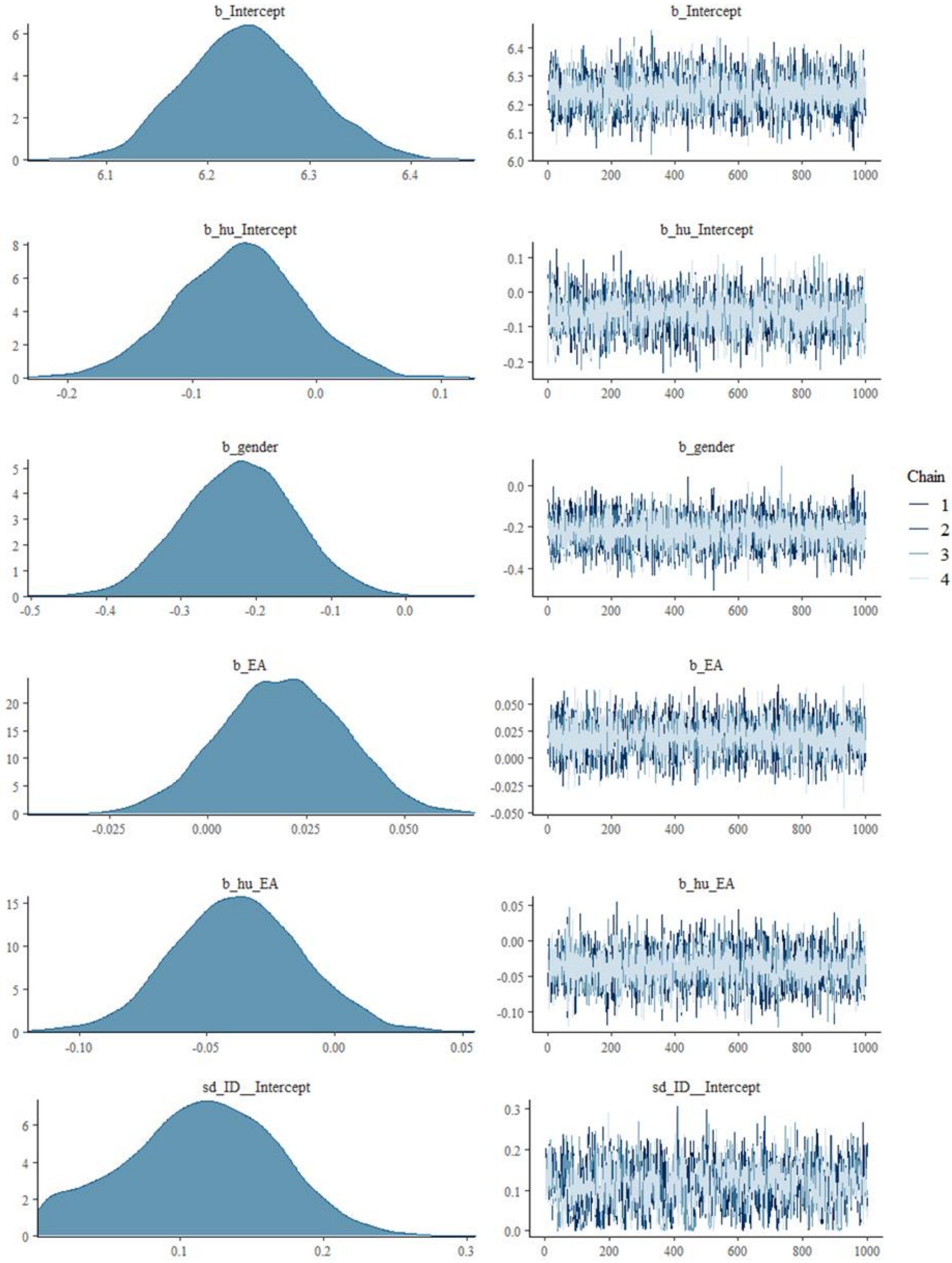

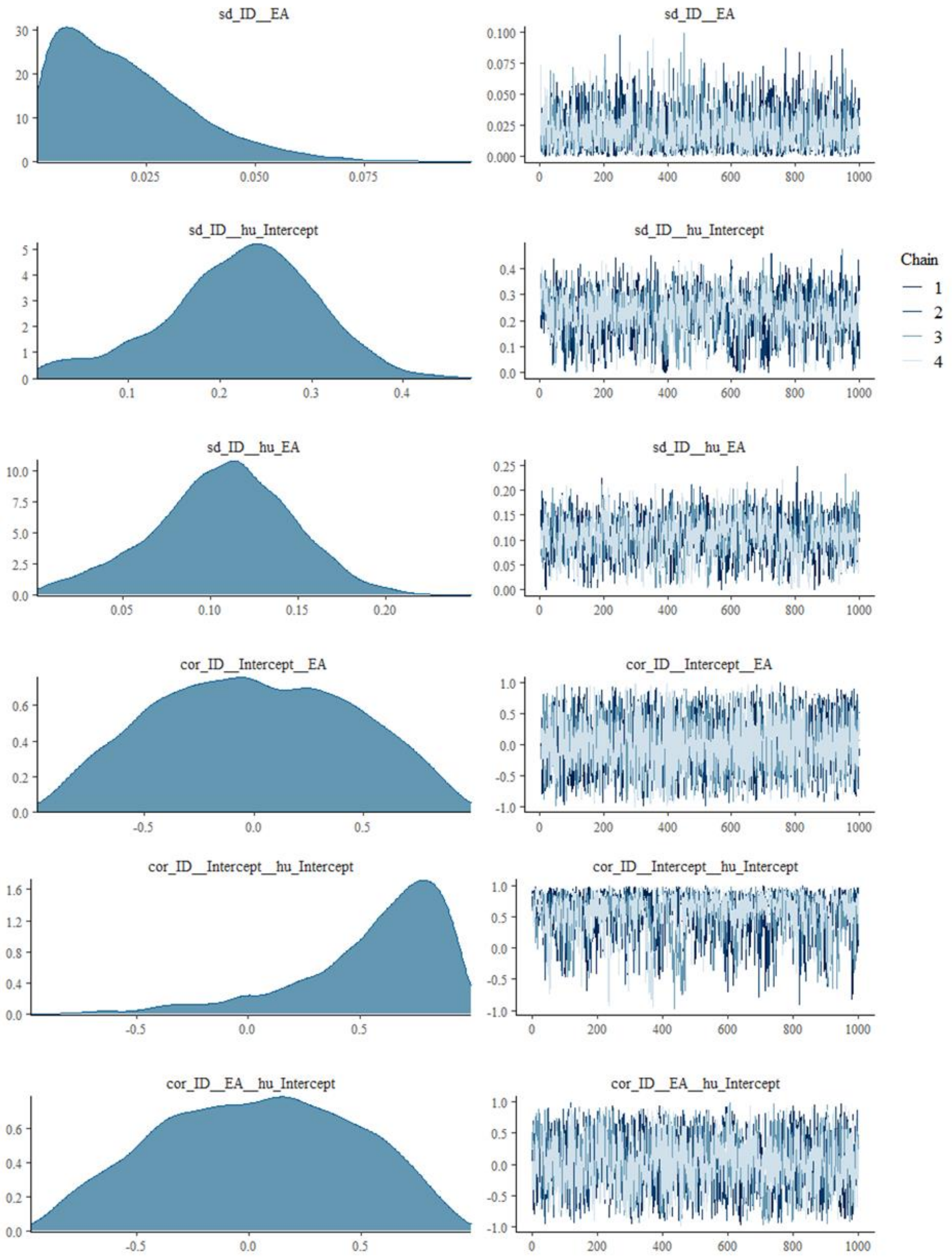

sd_ID__EA
sd_ID__hu_Intercept
sd_ID__hu_EA
cor_ID__Intercept__EA
cor_ID__Intercept__hu_Intercept
cor_ID__EA__hu_Intercept
Chain
1
2
3
4

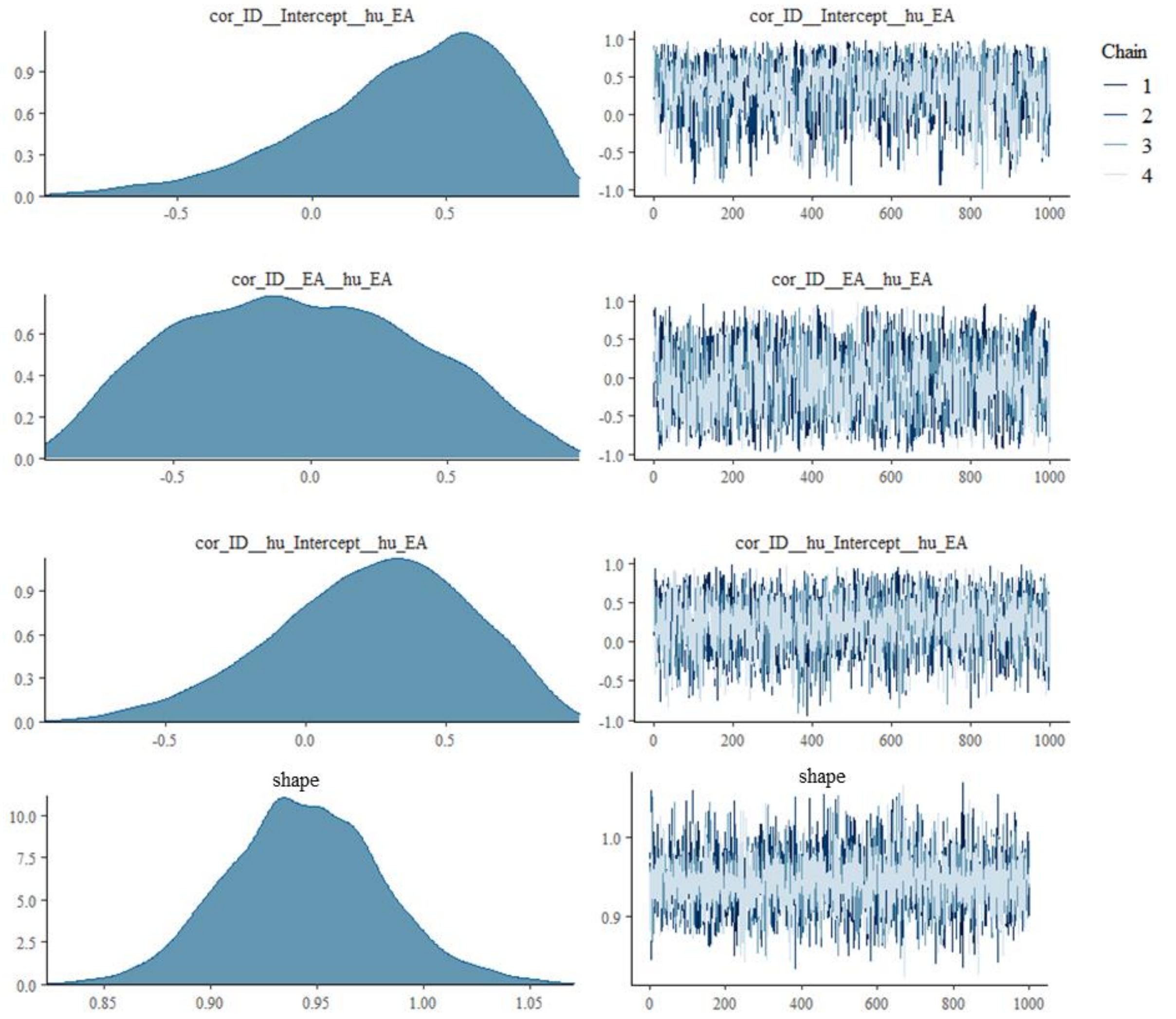
cor_ID__Intercept__hu_EA
cor_ID__Intercept__hu_EA
Chain
1
2
3
4
cor_ID__EA__hu_EA
cor_ID__EA__hu_EA
cor_ID__hu_Intercept__hu_EA
cor_ID__hu_Intercept__hu_EA
shape
shape
