# Supplementary material for: Studying dietary intake in daily life through multilevel two-part modelling: a novel analytical approach and its practical application
Source: Int J Behav Nutr Phys Act. 2021 Sep 27;18:130. doi: 10.1186/s12966-021-01187-8 (PMC8477527; doi:10.1186/s12966-021-01187-8)
Supplement: Supplementary file 6 — Additional file 6: Model Summaries. Figure 1. Model summary of the Random Intercept Model with Level-2 predictor gender. Figure 2. Model summary of the Random Slope Model with Level-1 predictor energetic arousal (EA). [file 12966_2021_1187_MOESM6_ESM.pdf]

Additional file 6. Model Summaries

Figure 1. Model summary of the Random Intercept Model with Level-2 predictor *gender*

```
Family: hurdle_gamma
  Links: mu = log; shape = identity; hu = logit
Formula: energy_intake ~ 1 + gender + (1 | x | ID)
         hu ~ 1 + gender + (1 | x | ID)
   Data: data (Number of observations: 2044)
Samples: 4 chains, each with iter = 2000; warmup = 1000; thin = 1;
         total post-warmup samples = 4000

Group-Level Effects:
~ID (Number of levels: 99)
                           Estimate  Est.Error l-95% CI u-95% CI Rhat Bulk_ESS Tail_ESS
sd(Intercept)                0.12 ($\sqrt{\sigma^2_{u_1}}$) 0.05     0.01     0.22  1.00     795    599
sd(hu_Intercept)             0.23 ($\sqrt{\sigma^2_{u_0}}$) 0.08     0.04     0.38  1.00     831    507
cor(Intercept,hu_Intercept)  0.71 ($\rho_{u_1 u_0}$) 0.34    -0.34     0.99  1.00     704    627

Population-Level Effects:
                Estimate   Est.Error  l-95% CI u-95% CI Rhat Bulk_ESS Tail_ESS
Intercept         6.27 ($\beta_{10}$)   0.06      6.14     6.39   1.00     4889     3135
hu_Intercept     -0.05 ($\beta_{00}$)   0.09     -0.23     0.13   1.00     4074     2942
gender           -0.25 ($\beta_{11}$)   0.07     -0.39    -0.10   1.00     4855     3059
hu_gender        -0.02 ($\beta_{01}$)   0.11     -0.24     0.20   1.00     4241     2749

Family Specific Parameters:
      Estimate Est.Error l-95% CI u-95% CI Rhat Bulk_ESS Tail_ESS
shape     0.94      0.04     0.87     1.02 1.00     5706     2843

Samples were drawn using sampling(NUTS). For each parameter, Bulk_ESS
and Tail_ESS are effective sample size measures, and Rhat is the potential
scale reduction factor on split chains (at convergence, Rhat = 1).
```

Figure 2. Model summary of the Random Slope Model with Level-1 predictor energetic arousal (*EA*)

```
Family: hurdle_gamma
  Links: mu = log; shape = identity; hu = logit
Formula: energy_intake ~ 1 + gender + EA + (1 + EA | x | ID)
         hu ~ 1 + EA + (1 + EA | x | ID)
   Data: data (Number of observations: 2025)
Samples: 4 chains, each with iter = 2000; warmup = 1000; thin = 1;
         total post-warmup samples = 4000

Group-Level Effects:
~ID (Number of levels: 99)
```

	Estimate		Est.Error	l-95% CI	u-95% CI	Rhat	Bulk_ESS	Tail_ESS
sd(Intercept)	0.11	$(\sqrt{\sigma^2_{u_1}})$	0.05	0.01	0.21	1.00	801	611
sd(EA)	0.02	$(\sqrt{\sigma^2_{u_{12}}})$	0.02	0.00	0.06	1.00	2144	1789
sd(hu_Intercept)	0.22	$(\sqrt{\sigma^2_{u_0}})$	0.08	0.04	0.37	1.00	1098	950
sd(hu_EA)	0.11	$(\sqrt{\sigma^2_{u_{01}}})$	0.04	0.02	0.18	1.00	1154	1608
cor(Intercept,EA)	0.00	$(\rho_{u_1u_{12}})$	0.45	-0.80	0.81	1.00	4486	2421
cor(Intercept,hu_Intercept)	0.56	$(\rho_{u_1u_0})$	0.34	-0.36	0.95	1.00	843	899
cor(EA,hu_Intercept)	0.04	$(\rho_{u_{12}u_0})$	0.43	-0.78	0.80	1.00	1744	2524
cor(Intercept,hu_EA)	0.36	$(\rho_{u_1u_{01}})$	0.37	-0.51	0.89	1.00	1177	1124
cor(EA,hu_EA)	-0.05	$(\rho_{u_{12}u_{01}})$	0.44	-0.81	0.78	1.00	1463	2423
cor(hu_Intercept,hu_EA)	0.24	$(\rho_{u_0u_{01}})$	0.34	-0.50	0.82	1.00	2347	2418

Population-Level Effects:

	Estimate		Est.Error	l-95% CI	u-95% CI	Rhat	Bulk_ESS	Tail_ESS
Intercept	6.24	(β_{10})	0.06	6.12	6.36	1.00	4883	3222
hu_Intercept	-0.06	(β_{00})	0.05	-0.16	0.04	1.00	4046	2537
gender	-0.22	(β_{11})	0.07	-0.37	-0.08	1.00	4479	2810
EA	0.02	(β_{12})	0.02	-0.01	0.05	1.00	5552	3172
hu_EA	-0.04	(β_{01})	0.03	-0.09	0.01	1.00	5232	3301

Family Specific Parameters:

	Estimate	Est.Error	l-95% CI	u-95% CI	Rhat	Bulk_ESS	Tail_ESS
shape	0.94	0.04	0.88	1.02	1.00	5073	2594

```
Samples were drawn using sampling(NUTS). For each parameter, Bulk_ESS
and Tail_ESS are effective sample size measures, and Rhat is the potential
scale reduction factor on split chains (at convergence, Rhat = 1).
```
